# Statin effects on the lipidome: Predicting statin usage and implications for cardiovascular risk prediction

**DOI:** 10.1016/j.jlr.2025.100800

**Published:** 2025-04-10

**Authors:** Changyu Yi, Kevin Huynh, Yvette Schooneveldt, Gavriel Olshansky, Amy Liang, Tingting Wang, Habtamu B. Beyene, Aleksandar Dakic, Jingqin Wu, Michelle Cinel, Natalie A. Mellett, Gerald F. Watts, Joseph Hung, Jennie Hui, John Beilby, Joanne E. Curran, John Blangero, Eric K. Moses, John Simes, Andrew M. Tonkin, Leonard Kritharides, David Sullivan, The LIPID Study Investigators, A. Tonkin, P. Aylward, D. Colquhoun, P. Glasziou, P. Harris, D. Hunt, A. Keech, S. MacMahon, P. Magnus, D. Newel, P. Nestel, N. Sharpe, J. Shaw, R.J. Simes, P. Thompson, A. Thomson, M. West, H. White, A. Thomson, S. Simes, D. Colquhoun, W. Hague, S. MacMahon, R.J. Simes, R.J. Simes, P. Glasziou, S. Caleo, J. Hall, A. Martin, S. Mulray, P. Barter, L. Beilin, R. Collins, J. McNeil, P. Meier, H. Willimott, P. Harris, W. Hague, D. Smithers, A. Tonkin, P. Wallace, H. Willimott, D. Hunt, J. Baker, P. Aylward, P. Harris, M. Hobbs, P. Thompson, N. Sharpe, D. Hunt, M. West, P. Thompson, H. White, P. Aylward, D. Colquhoun, D. Sullivan, A. Keech, P. Thompson, S. MacMahon, A. Tonkin, M. West, H. White, N. Anderson, G. Hankey, R.J. Simes, S. Simes, J. Watson, R.J. Simes, N. Sharpe, A. Thomson, A. Tonkin, H. White, W. Hague, J. Baker, M. Arulchelvam, S. Chup, J. Daly, J. Hanna, A. Leach, M. Lee, J. Loughhead, H. Lundie-Jenkin, J. Morrison, A. Martin, S. Mulray, S. Netting, A. Nguyen, H. Pater, R. Philip, G. Pinna, D. Rattos, S. Ryerson, V. Sazhin, S. Simes, R. Walsh, A. Keech, R.J. Simes, A. Clague, M. Mackie, J. Yallop, K. Boss, S. MacMahon, M. Whiting, M. Shepard, J. Leach, M. Gandy, J. Joughin, J. Seabrook, R. Abraham, J. Allen, F. Bates, I. Beinart, E. Breed, D. Brown, N. Bunyan, D. Calvert, T. Campbell, D. Condon-Paoloni, B. Conway, L. Coupland, J. Crowe, N. Cunio, B. Cuthbert, N. Cuthbert, S. D’Arcy, P. Davidson, B. Dwyer, J. England, C. Friend, G. Fulcher, S. Grant, K. Hellestrand, M. Kava, L. Kritharides, D. McGill, H. McKee, A. McLean, M. Neaverson, G. Nelson, M. O’Neill, C. Onuma, F. O’Reilly, A. Owensby, D. Owensby, J. Padley, G. Parnell, S. Paterson, C. Pawsey, R. Portley, K. Quinn, D. Ramsay, M. Russell, J. Ryan, B. Sambrook, L. Shields, J. Silberberg, S. Sinclair, D. Sullivan, P. Taverner, D. Taylor, M. Taylor, M. Threlfall, J. Turner, A. Viles, J. Waites, R. Walker, W. Walsh, K. Wee, P. West, R. Wikramanayake, D. Wilcken, J. Woods, R.K. Barnett, Z. Bogetic, H. Briggs, A. Broughton, L. Brown, A. Buncle, P. Calafiore, L. Carrick, Y. Cavenett, L. Champness, R. Clark, H. Connor, J. Counsell, J. Deague, G. Derwent-Smith, A. Driscoll, B. Feldtmann, L. Fisher, B. Forge, A. Hamer, H. Harrap, S. Hodgens, M. Hooten, J. Hurley, B. Jackson, J. Johns, J. Krafchek, H. Larwill, I. Lyall, S. Marks, M. Martin, B. Mason, J. McCabe, C. Medley, L. Morgan, L. Mullan, D. Ogilvy, G. Phelps, P. Phillips, H. Prendergast, D. Rose, G. Rudge, W. Ryan, M. Sallaberger, G. Savige, B. Sia, A. Soward, C. Steinfort, K. Tankard, J. Tippett, B. Tyack, J. Voukelatis, M. Wahlqvist, N. Walker, S. Whitten, R. Yee, M. Zanoni, R. Ziffer, K. Anderson, G. Aroney, C. Atkinson, K. Boyd, R. Bradfield, G. Cameron, D. Careless, A. Carle, P. Carroll, T. Carruthers, D. Chaseling, B. Cooke, S. Coverdale, B. Currie, M. d’Emden, F. Ekin, R. Elder, T. Elsley, L. Ferry, C. Gnanaharan, K. Graham, K. Gunawardane, C. Hadfield, C. Halliday, R. Halliday, A. Heyworth, B. Hicks, P. Hicks, T. Htut, L. Hughes, J. Humphries, H. LeGood, J. Nye, D. O’Brien, G. Real, K. Roberts, L. RossLee, J. Sampson, I. Scott, H. Smith, V. Smith-Orr, Y. Tan, B. Wicks, J. Wicks, S. Woodhouse, J. Bradley, L. Callaway, A. Calvert, J. Crettenden, A. Dufek, B. Dunn, C. Dunphy, D. Gow, I. Hamilton-Craig, K. Herewane, S. Keynes, L. McLeay, R. McLeay, L. Ng, C. Thomas, P. Tideman, L. Wilson, R. Yeend, C. Zhang, Y. Zhang, P. Bradshaw, M. Brooks, R. Burton, J. Garrett, K. Gotch-Martin, J. Hargan, B. Hockings, G. Lane, S. Ross, R. Cutforth, D. D’Silva, W. Hitchener, V. Kimber, G. Kirkland, P. Neid, R. Parkes, B. Singh, C. Singh, M. Smith, S. Smith, M. Templer, N. Whitehouse, R. Allen-Narker, R. Anandaraja, S. Anandaraja, P. Barclay, S. Baskaranathan, P. Bridgman, J. Brown, J. Bruning, J. Calton, A. Clague, M. Clark, D. Clarke, T. Cook, R. Coxon, M. Denton, A. Doone, R. Easthope, J. Elliott, C. Ellis, P. FosterPratt, C. Frenneux, M. Frenneux, D. Friedlander, D. Fry, L. Gibson, M. Gluyas, A. Hall, K. Hall, A. Hamer, H. Hart, P. Healy, J. Hedley, P. Heuser, H. Ikram, D. Jardine, J. Kenyon, H. King, T. Kirk, T. Lawson, P. Leslie, G. Lewis, E. Low, R. Luke, S. Mann, D. McClean, D. McHaffie, L. Nairn, H. Patel, L. Pearce, K. Ramanathan, R. Rankin, J. Reddy, S. Reuben, R. Ronaldson, D. Roy, H. Roy, P. Scobie, D. Scott, J. Scott, K. Skjellerup, R. Stewart, D. Walters, T. Wilkins, A. Vitanachy, P. Wright, A. Zambanini, Jonathan E. Shaw, Dianna J. Magliano, Agus Salim, Corey Giles, Peter J. Meikle

**Affiliations:** 1 Diabetes and Population Health, Baker Heart and Diabetes Institute, Melbourne, Australia; 2 Baker Department of Cardiometabolic Health, Melbourne University, Melbourne, Australia; 3 Baker Department of Cardiovascular Research Translation and Implementation, La Trobe University, Melbourne, Australia; 4 Faculty of Medicine, Nursing and Health Sciences, Monash University, Melbourne, Australia; 5 School of Medicine, University of Western Australia, Perth, Australia; 6 Lipid Disorders Clinic, Department of Cardiology, Royal Perth Hospital, Perth, Australia; 7 School of Biomedical Sciences, University of Western Australia, Perth, Australia; 8 School of Population and Global Health, University of Western Australia, Perth, Australia; 9 South Texas Diabetes and Obesity Institute, The University of Texas Rio Grande Valley, Brownsville, TX, USA; 10 Menzies Institute for Medical Research, University of Tasmania, Hobart, Australia; 11 National Health and Medical Research Council of Australia (NHMRC), Clinical Trials Centre, University of Sydney, Sydney, Australia; 12 School of Public Health and Preventive Medicine, Monash University, Melbourne, Australia; 13 Atherosclerosis and Vascular Biology Laboratory, ANZAC Medical Research Institute, Sydney, Australia; 14 Concord Repatriation General Hospital, Sydney Local Health District, Sydney, Australia; 15 New South Wales Health Pathology, Sydney, Australia; 16 Clinical Diabetes and Epidemiology, Baker Heart and Diabetes Institute, Melbourne, Australia; 17 Melbourne School of Population and Global Health and School of Mathematics and Statistics, The University of Melbourne, Melbourne, Australia

**Keywords:** lipidomics, cardiovascular disease, statins, regularized linear models

## Abstract

Statin therapy is a highly successful and cost-effective strategy for the prevention and treatment of cardiovascular diseases (CVD). Adjusting for statin usage is crucial when exploring the association of the lipidome with CVD to avoid erroneous conclusions. However, practical challenges arise in real-world scenarios due to the frequent absence of statin usage information. To address this limitation, we demonstrate that statin usage can be accurately predicted using lipidomic data. Using three large population datasets and a longitudinal clinical study, we show that lipidomic-based statin prediction models exhibit high prediction accuracy in external validation. Furthermore, we introduce a re-weighted model, designed to overcome a ubiquitous limitation of prediction models, namely the need for predictor alignment between training and target data. We demonstrated that the re-weighted models achieved comparable prediction accuracy to ad hoc models which use the aligned predictor between training and target data. This innovation holds promise for significantly enhancing the transferability of statin prediction and other ‘omics prediction models, especially in situations where predictor alignment is incomplete. Our statin prediction model now allows for the inclusion of statin usage in lipidomic analyses of cohorts even where statin use is not available, improving the interpretability of the resulting analyses.

Statins and lipid-lowering medications have made a tremendous impact on the prevention of CVD events (1, 2, 3). Statins inhibit the rate-limiting enzyme in cholesterol biosynthesis—HMG-CoA reductase (HMGCR)—which leads to reduced circulating low density lipoprotein cholesterol (LDL-C) levels which is a risk factor of CVD. Statin treatment and LDL-C reduction have shown considerable risk reduction from CVD in primary (4, 5) and secondary prevention (3). Despite our progress in understanding CVD risk factors and an expanding range of preventative therapies, a significant number of individuals progress to acute CVD. Therefore, there is a need to continue elucidating the molecular mechanisms and pathophysiology of CVD.

An accumulating body of evidence has demonstrated that lipid metabolism—beyond cholesterol metabolism—is actively involved in the etiology, progression, and sequelae of CVD (6, 7). Lipidomic risk scores, encompassing diverse lipid species, independently predict incident CVD even after accounting for traditional risk factors (8, 9, 10). Furthermore, changes in lipid metabolism following statin treatment have been shown to predict an individual’s risk reduction from treatment (6). This statin-lipid metabolism interaction is an important consideration for studies aiming to identify biomarkers of CVD. Adjustment for statins and other lipid-lowering medications is a crucial component of any rigorous investigation. However, many prospective studies are hampered by a lack of or poor quality information about medication usage.

In this study, we uncover the effect of statin treatment on the lipidome using a large randomized clinical trial (n = 4,991, measured before and 12 months after statin commencement). Using a large population-based cohort (n = 10,339), we demonstrate the bias that can occur in metabolomic investigations when statin usage is omitted from analysis—a large increase in false positive associations and inflation in odds ratios is evident, which can lead to incorrect conclusions about molecular mechanisms. Using a combined total of 24,194 individuals, we demonstrate that statin usage can be robustly predicted using lipidomic profiles. To overcome the issue of incomplete overlap in lipidomic measurements between studies, we present a novel re-weighted transfer method to improve the transferability of prediction models. The re-weighted transfer method allows models to be used in datasets missing a large number of variables without substantial loss of prediction accuracy. We demonstrate that the re-weighted model shows comparable prediction accuracy in external validation to ad hoc ridge models. Code to develop and use re-weighted transfer models is available as an R package (Flexible Transfer Models, FTM).

## Materials and Methods

### Ethical approval

The Australian Diabetes, Obesity and Lifestyle Study (AusDiab) was approved by the Alfred Human Research Ethics Committee, Melbourne, Australia (project approval number, 41/18). The Long-term Intervention with Pravastatin in Ischemic Disease trial (LIPID) biobank was also approved by the Alfred Human Research Ethics Committee (project approval number, 85/11 and 376/22). The Busselton Health Study (BHS) cohort was approved by the University of Western Australia Human Research Ethics Committee (approval number 608/15). The San Antonio Family Heart Study (SAFHS) was reviewed and approved by the Institutional Review Board at the University of Texas Rio Grande Valley (IRB-18-0245, IRB-18-0255, and IRB-18-0406). Informed consent was obtained from all participants, and studies were conducted in accordance with the ethical principles of the Declaration of Helsinki. No participant compensation was provided.

### Cohort descriptions

AusDiab was conducted from 1999 to 2012 and is a nationwide prospective investigation focusing on diabetes and CVD within the Australian adult population (11). The baseline survey encompassed 11,247 participants, selected through a stratified cluster sampling method from urban and rural regions in all states across Australia. After the exclusion of participants who were pregnant, those with missing data, or due to technical issues related to lipidomic analysis (very low/no signal detected), baseline fasting plasma samples from 10,339 individuals were analyzed. The study employed established measurement techniques for clinical lipids, including fasting serum total cholesterol, high-density lipoprotein cholesterol (HDL-C), and triglycerides, as well as anthropometric measurements like height, weight, and body mass index (BMI) (11, 12). Prevalent CVD cases in the study include prior heart attacks, strokes, and angina.

BHS is an independent population-based study conducted in the town of Busselton, Western Australia. The study participants primarily consisted of individuals of white/European origin. The specific dataset used for validation included 4,492 subjects from the 1994/95 survey, which was part of an ongoing epidemiological investigation. The mean age of BHS is 50.8 years, and women constitute 56% (2,516 individuals) of the total cohort. The study's design and measurements of HDL-C, LDL-C, triglycerides, total cholesterol, and BMI have been documented in previous publications (13, 14, 15).

SAFHS is a pedigree-based prospective cohort study, designed to investigate the genetic and environmental factors influencing cardiovascular risk factors in Mexican Americans. In 1991, SAFHS enrolled 42 extended Mexican American families living in San Antonio, Texas. Probands were selected at random with no enrichment for disease. The study's enrollment procedures, inclusion and exclusion criteria, and the detailed phenotypic assessments of participants have been previously documented (16, 17). This ongoing study encompasses multiple phases of data collection and recruitment, involving 2,595 individuals, including first-, second-, and third-degree relatives of the proband and the proband's spouse, as well as spouses of these relatives.

LIPID study was a randomized, placebo-controlled trial conducted across 87 centers in Australia and New Zealand, as originally described in previous publications (3, 18). It involved 9,014 participants who had experienced an acute myocardial infarction or received a hospital diagnosis of unstable angina pectoris 3–36 months prior, with total cholesterol levels ranging from 4.0 to 7.0 mmol/L. Participants were randomly assigned to receive either pravastatin (40 mg daily) or a placebo, in addition to their standard treatment, dietary advice, and care provided by their physicians. Plasma samples and clinical lipid measurements were collected at baseline and one year following randomization. We performed a detailed fasting plasma lipidomic analysis on 6,782 participants, among whom 1,000 had an available sample from baseline only, 791 had a sample at 12 months only, and 4,991 had samples at both baseline and 12 months. A total of 45 lipidomic sample outliers were identified and subsequently excluded from the LIPID dataset, involving 42 participants.

### Lipid extraction and liquid chromatography mass spectrometry

Given the large number of samples in each cohort, we considered factors such as extraction efficiency, lipid stability, cost, and throughput when selecting a lipid extraction method. We chose a single-phase butanol/methanol approach, which has been shown to provide a good balance of these factors, as previously described. (19, 20). In brief, 10 μl of plasma (AusDiab, LIPID, SAFHS) or serum (BHS) was mixed with 100 μl of butanol/methanol (1:1) containing 10 mM ammonium formate and a standard mix of internal standards. Samples were vortexed thoroughly, followed by sonication for 60 min at room temperature. Each sample was subsequently centrifuged (14,000 *g*, 10 min), and the supernatant containing the lipid extract was collected and stored at −80°C prior to analysis.

Targeted lipidomic analysis was performed by electrospray ionization-tandem mass spectrometry using a triple quadrupole mass spectrometer with an Agilent 1,290 series high-performance liquid chromatography (HPLC) system as described previously for AusDiab (12, 21), BHS (22), SAFHS (23), and LIPID (8). A 1 μl injection was used for each sample, and mass spectrometry analysis was performed using dynamic scheduled multiple reaction monitoring (21). AusDiab and SAFHS utilized positive/negative ion mode switching, while BHS and LIPID were run in just positive ion mode.

Lipid concentrations were calculated by relating the area under the chromatographic peak to its corresponding internal standard. Correction factors were used to adjust for differences in response, where these were known. For a comprehensive overview of the lipidomic method, see (21, 24).

### Harmonization of lipidomic datasets

To harmonize lipidomic datasets, we established consensus lipid concentrations for the National Institute of Standards and Technology-Standard Reference Material-1950 (NIST-SRM-1950) samples. Concentrations from a total of 1,017 unique NIST-SRM-1950 samples were combined. These studies were profiled over five years, across 24 independent studies, and on six mass spectrometers, thereby encompassing a wide range of analytical conditions. Using this dataset, we calculated the reference point concentrations for 932 lipid species from 50 lipid classes (supplemental Table S1). It is important to note that these concentrations do not represent 'absolute concentrations' per se. Rather, they serve as a reliable reference point—derived from a large collection of independent measurements—to facilitate the harmonization of lipidomic datasets.

To correct misalignment of concentrations between datasets, we applied a correction factor to each lipid species (supplemental Table S2). This correction factor was determined as the ratio of the reference point concentration of NIST-SRM-1950 to the median concentration of NIST-SRM-1950 measured in the specific cohort. The AusDiab and SAFHS cohorts had NIST-SRM-1950 interspersed with cohort samples, which allowed direct alignment. The BHS and LIPID studies, on the other hand, did not measure NIST-SRM-1950 at the time of profiling. To overcome this challenge, we created matched sub-cohorts between BHS/LIPID and AusDiab, based on study-appropriate covariates (age, sex, BMI, total cholesterol, HDL-C, triglycerides; BHS-specific: absence of prevalent disease; LIPID-specific: prevalent-CVD). Correction factors were then determined as the ratio of the median AusDiab sub-cohort to the median BHS/LIPID sub-cohort. This method ensures that the lipid concentrations in BHS and LIPID were effectively harmonized with AusDiab and SAFHS despite the absence of NIST-SRM-1950 measurements in these cohorts (25).

The lipidomic profiling methodology has evolved over time, as such, each cohort was profiled with different levels of granularity. To overcome this issue, we used a recently published approach to impute unmeasured high-resolution lipid species into a lower-resolution dataset using a reference dataset (26). Using AusDiab as the reference dataset, 160 and 410 unmeasured lipid species were imputed in BHS and LIPID, respectively. Only imputed lipid species of high quality (expected correlation with true measurement > 0.6) were kept (26).

### Statin usage information of the cohorts

Baseline statin usage in the AusDiab cohort was derived from the questionnaire: “are you currently taking tablets to lower your cholesterol/triglycerides?”. At the 5-year follow-up, detailed medication information was collected from participants, including the brand and dose. In the LIPID trial, all participants were not taking any other lipid-lowering medication at baseline, as part of the run-in phase. 49.0% of patients were later randomized to pravastatin treatment (40 mg), after the baseline blood samples were collected. The follow-up samples were taken 12 months after randomization. In BHS, statin usage was derived from self-reported medication usage. In SAFHS, all participants were instructed to bring medications to the interviews, which were recorded.

### Assessing the effect of statin use on lipid concentrations

The association of lipid species and classes with statin usage was assessed in the LIPID cohort using linear regression, mainly as LIPID contains the baseline and 12-month follow-up lipidomic data. Only individuals with both baseline and 12-month follow-up measurements were included in the analysis. The linear model involved modeling 12-month follow-up lipid measurements against statin usage, while adjusting for baseline concentrations and covariates such as age, sex, BMI, hypertension medication, diabetes, current smoking status, and blood pressure. Interactions between baseline lipid concentration and statin usage were also considered in the model. To further evaluate the effects of statin use on lipid metabolism independent of lipoprotein changes, we performed a separate regression adjusting for 12-month total cholesterol, HDL-C, and triglycerides.

### Definition of previous cardiovascular disease

Previous cardiovascular disease in the AusDiab cohort was based on combining responses to question “have you been told you have had a heart attack/stroke/angina?”. All the patients involved in the LIPID trial had a cardiovascular event as the main criterion for recruitment (acute myocardial infarction or unstable angina pectoris), often accompanied with claudication (10%), stroke (4%), and angina pectoris (37%). In SAFHS, major adverse cardiac events include heart attack, stroke, and death from an event. In BHS, prevalent ischaemic heart disease was defined through health-linkage (International Classification of Diseases-10 codes I20-I25).

### Association of lipid species with prevalent cardiovascular disease

The association of lipid species with prevalent CVD was examined in the AusDiab cohort using logistic regression. To demonstrate the impact of statin adjustment, two separate models were employed, one including statin usage as a covariate and the other without. All models were adjusted for covariates, including age, sex, BMI, hypertension medication, current smoking status, and systolic blood pressure. Associations were quantified and reported as log odds ratios along with standard errors.

### Creation of a statin prediction model using lipidomic data

Lipidomic data were log-transformed before statistical analysis. Regularized (L_2_) logistic regression ridge models were created using glmnet (27), using lipid species in common between the training and validation cohorts. For the predictive model, lipid species were treated as predictors, and statin usage was the outcome, while adjusting for age and sex. Cross-validation (10-fold) was used to identify the optimal amount of regularization (lambda), then a final model was produced using all samples and the lambda that gave the lowest cross-validated deviance. Note that when using the LIPID lipidomics data to create prediction models, the imputed lipid species were not used to avoid rank deficiency in the design matrix.

### Creation of a flexible transfer model for statin prediction

Despite the harmonization of lipidomic datasets, each dataset may still contain a different number of lipid species. To circumvent the need for cohort-specific models, we introduce a flexible structure to represent the model. The probability that an outcome *Y* equals a certain class, in a standard logistic regression, is given by:

$$P\left(Y=1|X\right)=\frac{1}{1+e^{-\left(\beta_{0}+\beta_{1}X_{1}+\beta_{2}X_{2}+\cdots +\beta_{n}X_{n}\right)}}$$

where $X=\left\{X_{1},X_{2},\ldots ,X_{n}\right\}$ represents the predictor variables, and $\beta =\left(\beta_{0},\beta_{1},\ldots \beta_{n}\right)$ are the coefficients to be estimated. The coefficients can be estimated through an Iteratively Reweighted Least Squares (IRLS) procedure. Following the convergence of the IRLS procedure, the beta coefficients can be calculated as:

$$\hat{\beta }={\left(X^{T}WX\right)}^{-1}X^{T}Wz$$

In this equation, W is a diagonal matrix of weights, where diagonal elements *W*_*ii*_ is calculated as $W_{ii}=p_{i}\left(1-p_{i}\right)$, where *p*_*i*_ is the predicted probability for the *i*-th individual. *z* is a vector representing the log odds for all individuals, calculated as the logit of the predicted probabilities ($z_{i}=\log\left(\frac{p_{i}}{1-p_{i}}\right)$). Following this, we designate $X^{T}WX$ as *M* and $X^{T}Wz$ as υ. Therefore, we can estimate the beta coefficients for the full variable set as $\hat{\beta }={\left(M\right)}^{-1}\upsilon$.

To adapt the model to a reduced variable set, we define *X*_*reduced*_ as a subset of the original variables:

$$X_{reduced}=\left\{X_{j}|j\in J\right\}$$

where J⊆{1,2,…,n} is an index set that specifies which variables from *X* are included in *X*_*reduced*_. The matrix $M_{reduced}$ can be obtained by extracting the submatrix $M_{J,J}$ from M. Similarly, the vector $\upsilon_{reduced}$ is composed of rows of υ that are indexed by *J*. Estimation of beta coefficients for the reduced variable set can be approximated as:

$${\hat{\beta }}_{reduced}=M_{reduced}^{-1}\upsilon_{reduced}$$

This new formula will provide an excellent approximation to the estimated beta coefficients in a model with *X*_*reduced*_ as independent variables, as long as the predicted probabilities with and without the excluded variables do not change much or when they change, the changes are proportional across all individuals, so that the ratio of predicted probabilities for any two individuals are relatively similar in the two models.

This approximation provides a novel approach to re-estimate beta coefficients for the reduced variable sets, without the need for re-optimization or access to the individual-level data. By storing and distributing the M and υ matrices, rather than just beta coefficients, models can adapt and be applied to datasets with a differing number of variables, substantially enhancing the model’s versatility and applicability across different datasets.

### Robustness assessment of the flexible transfer models

To assess the robustness of the flexible transfer model (FTM), we compared its predictive performance against cohort-specific ridge models in external datasets. We evaluated the performance in external datasets, simulating the situation where only a subset of lipids were available. Specifically, we selected a random subset of lipids, from all lipids (100%) down to only 10% of lipid species, in increments of 10%. For each increment, we resampled the subset of lipids 10 times to simulate different dataset scenarios. For each resample, we tested the performance of the FTM against a ridge model built for the corresponding cohort. Beta-coefficients that no longer had corresponding lipid measurements (due to selecting a subset of lipids) were discarded from the ridge model calculations. To demonstrate that the FTM provides accurate performance for the subsets, ridge models were built using the lipid subsets, and the performance of the newly built ridge model was compared with the FTM in external cohorts.

## Results

### Lipidomic profiling of the cohorts used in this study

AusDiab and BHS represent longitudinal population studies conducted in Australian adults, while the LIPID trial is a randomized clinical study to investigate the efficacy of pravastatin in reducing the risk of cardiac events. SAFHS is an extensive, pedigree-based, long-term genetic study aimed at identifying genes associated with CVD risk in Mexican Americans, which has spanned 27 years. Notably, statin (or lipid-lowering medication) usage was markedly different in each cohort, which can partially be explained by the nature of the cohort and the year that samples were collected—statin usage has continued to rise every year since their widespread availability (28). The proportion of individuals taking statins (at baseline) for each cohort was 8.4%, 2.4%, 1.8%, and 0.0% for AusDiab, BHS, SAFHS, and LIPID (prior to randomization), respectively. The baseline characteristics of participants from different studies are presented in Table 1.

**Table 1:** Anthropometric and clinical characteristics of the cohorts used in this study

Characteristic	AusDiab	BHS	SAFHS	LIPID
n	10,339	4,492	1,366	6,768
Age, mean (SD)	51.3 (14.3)	50.8 (17.4)	39.2 (16.9)	62.6 (8.4)
Sex, n (% female)	5,685 (55.0)	2,516 (56.0)	808 (59.2)	1,149 (17.0)
BMI (kg/m^2^), mean (SD)	27.0 (4.9)	26.2 (4.2)	29.2 (6.6)	26.8 (3.9)
Total cholesterol (mmol/L), mean (SD)	5.66 (1.07)	5.60 (1.10)	4.90 (1.02)	5.65 (0.82)
HDL (mmol/L), mean (SD)	1.43 (0.38)	1.39 (0.39)	1.29 (0.33)	0.95 (0.24)
LDL (mmol/L), mean (SD)	3.54 (0.94)	3.59 (0.96)	2.82 (0.84)	3.88 (0.75)
Triglycerides (mmol/L), mean (SD)	1.54 (1.07)	1.18 (0.90)	1.70 (1.46)	1.81 (0.92)
Statin usage, n (%)	871 (8.4)	108 (2.4)	24 (1.8)	3,368 (49.0)^a^
Current smoking, n (%)	1,623 (15.7)	608 (13.5)	316 (21.1)	625 (9.2)
Diabetes, n (%)	686 (6.6)	271 (6.0)	202 (14.8)	578 (8.5)
Prevalent cardiovascular disease, n (%)	577 (5.6)	238 (5.3)	39 (2.9)	6,768 (100)

a Statin usage after randomization.

The number of lipid species measured in each cohort varies due to iterative improvements to the Baker lipidomics platform. Our initial platform was used to characterize the LIPID trial (342 lipid species), followed by BHS (596 lipid species), AusDiab (747 lipid species), and lastly SAFHS (857 lipid species). The NIST-SRM-1950 human plasma sample was included in the lipidomic extraction and mass spectrometry analysis for our more recent studies (AusDiab and SAFHS) as additional quality control samples. After quality control procedures, four lipid species were excluded from AusDiab, and one from BHS.

### Effect of pravastatin treatment on plasma lipidome

**Fig. 1:**
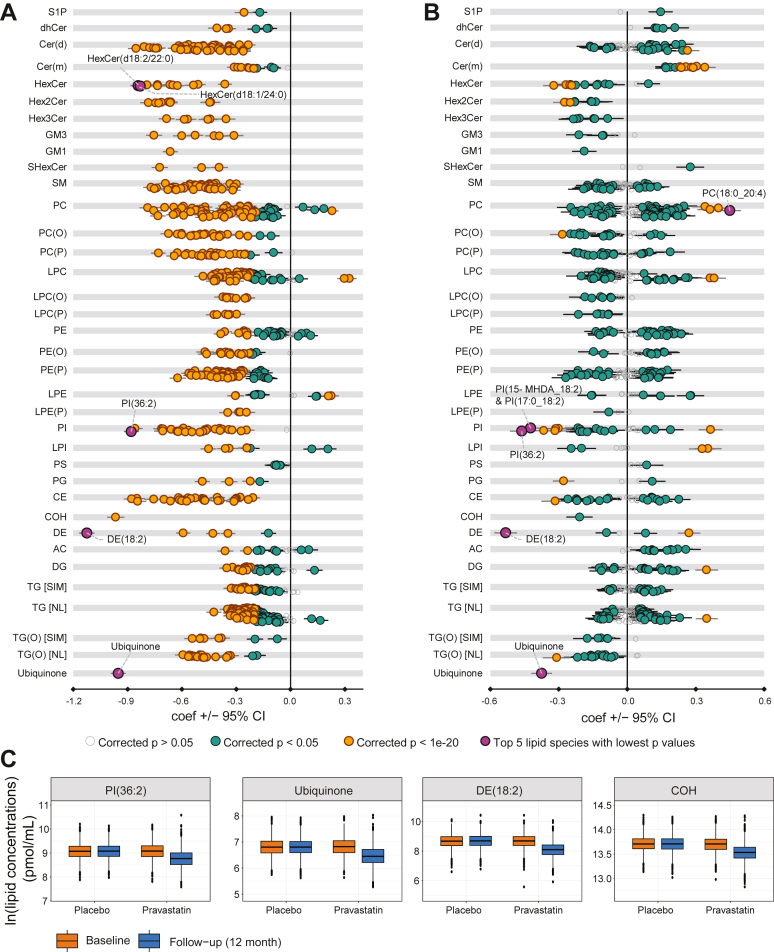
Association of statin usage with LIPID trial lipid species using linear regression. Linear regression analysis between each 12-months lipid species concentration and statin usage was performed while adjusting for age, sex, BMI, hypertension medication, current smoking, blood pressure, baseline lipid concentration, interaction between baseline lipid concentration, statin usage and clinical lipids (total cholesterol, HDL-C, triglycerides) from baseline (A) as well as clinical lipids from 12-months follow-up (B). (C) Boxplots (center line, median; box limits, upper and lower quartiles; vertical line, 1.5x interquartile range; points, outliers) showing natural log-transformed concentrations of top three negatively associated lipids and free cholesterol in baseline and 12-months follow-up. Grey empty circles show species with corrected *P* > 0.05, dark-green circles show species with corrected *P* < 0.05, yellow circles show species with corrected *p* < 1e-20, and purple circles represent top five species with lowest *P*-values. Whiskers represent 95% confidence intervals. The underlying data can be found in supplemental Table S3. AC, Acylcarnitine; CE, cholesteryl ester; Cer(d), ceramide; Cer(m), Deoxyceramide; COH, free cholesterol; DE, dehydrocholesterol; DG, diacylglycerol; dhCer, dihydroceramide; GM1, GM1 ganglioside; GM3, GM3 ganglioside; HexCer, monohexosylceramide; Hex2Cer, dihexosylceramide; Hex3Cer, trihexosylceramide; LPC, lysophosphatidylcholine; LPC(O), lysoalkylphosphatidylcholine; LPC(P), lysoalkenylphosphatidylcholine; LPE, lysophosphatidylethanolamine; LPE(P), lysoalkenylphosphatidylethanolamine; LPI, lysophosphatidylinositol; NL, neutral loss; PC, phosphatidylcholine; PC(O), alkylphosphatidylcholine; PC (P), alkenylphosphatidylcholine; PE, phosphatidylethanolamine; PE(O), alkylphosphatidylethanolamine; PE(P), alkenylphosphatidylethanolamine; PG, phosphatidylglycerol; PI, phosphatidylinositol; PS, Phosphatidylserine; SM, sphingomyelin; SIM, single ion monitoring; S1P, sphingosine-1-phosphate; SHexCer, Sulfatide; TG, triacylglycerol; TG(O), alkyl-diacylglycerol.

To examine the relationship between pravastatin treatment and the circulating concentration of lipid species, we conducted linear regression analyses in the LIPID cohort, using the 12-month lipid species concentrations as the outcome variables and statin treatment as the predictor (see details in the Materials and Methods section). We adjusted for covariates, including age, sex, BMI, use of hypertension medication, current smoking status, systolic blood pressure, baseline lipid concentration, baseline clinical lipids (total cholesterol, HDL, triglycerides), and the interaction between baseline lipid concentration and statin usage. Notably, statin usage was associated with lower concentrations in 35 out of 36 lipid classes after correcting multiple comparisons (q < 0.05; supplemental Fig. S1A). The only class that showed no significant change in response to pravastatin treatment was total acylcarnitine (supplemental Fig. S1A and supplemental Table S3). At the individual lipid species level, we observed that pravastatin usage was associated with lower concentrations in 683 out of 752 lipid species, including free cholesterol (β = −0.97, q = 7.59 × 10^-297^) (Fig. 1A and supplemental Table S3). Interestingly, we also identified 22 lipid species, from eight classes, and were significantly increased in response to pravastatin treatment. A majority of the lipids increasing in concentration contain arachidonic acid (20:4) side chains (13 out of the 22 lipid species increasing in concentration). The lipid species increasing the most was LPC (20:4) [sn1] (β = 0.33, q = 5.14 × 10^−57^; supplemental Table S3). We further investigated whether there was an interaction between pravastatin usage and the baseline concentration of each lipid species. Only two lipids showed a significant association for the interaction term after multiple comparison correction (supplemental Table S4). This indicates that the effect of pravastatin treatment on the concentration of lipid species is largely independent of baseline lipid concentrations.

Given the effect of statins on lipoprotein concentrations, we performed the same linear regression as above but adjusted for clinical lipids at the 12-months follow-up. The effect of this is to adjust for the overall decrease in LDL particles, and slight increase in HDL-particles resulting from the statin treatment so that we could define the change to the underlying lipid metabolism, independent of these lipoprotein effects. This approach also provides an indication of the qualitative changes in the lipoprotein composition and the results are independent of lipoprotein particle number. After this adjustment, we observed pravastatin usage was associated with higher concentrations in eight lipid classes, independent of changes in lipoproteins (supplemental Fig. S1B). Similar to previous results, 16 lipid classes showed decreases in concentrations after adjustment for multiple comparisons. After accounting for changes in lipoprotein concentrations, we only observed 476 lipid species showed significant changes in concentrations following pravastatin treatment (q < 0.05; Fig. 1B and supplemental Table S3), which is 229 less compared with the model without accounting for it. The lipid species most altered by pravastatin were DE (18:2) (β = −0.53, q = 3.23 × 10^−89^), PI (36:2) (β = −0.46, q = 1.93 × 10^−64^), and Ubiquinone (β = −0.37, q = 1.75 × 10^−56^) (Fig. 1C). Strikingly, the number of lipid species with a positive association with statin treatment increased from 22 to 237 after adjusting for clinical lipids at the 12-months follow-up, and 181 out of 237 lipid species showed negative associations with statin treatment when not considering adjusting 12-months follow-up clinical lipids (supplemental Table S3). In general, most lipid classes such as PE, PC, SM and CE show more positive associated lipid species compare with the previous model. Almost all lipid species from dhCer and Cer(m) classes showed negative association with statin treatment before taking account 12-months follow-up clinical lipids while they were positively associated with adjustment of that (Fig. 1A, B). It is also worth noting that all lipid species from some classes such as HexCer, LPC(O), LPC(P) and TG(O) still show negative associations with stain with lower *P* values, suggesting those lipids might be less affected by lipoproteins (Fig. 1A, B). Taking together, these results show that statin usage causes substantial changes to lipid metabolism beyond lowering LDL-C.

### Statin adjustment clarifies the association between lipids and prevalent CVD

Statin usage produces significant alteration in circulating lipid abundance while also reducing an individual’s risk of disease. Therefore, any analysis risks misinterpreting the direct impact of lipid species on CVD risk if statin usage is not properly accounted for. To explore this, we investigated the association between prevalent CVD and lipid species in the AusDiab cohort using two models: one without adjusting for statin use and the other with statin adjustment. Subsequently, we compared the odds ratios and *P*-values of the lipids obtained from both models.

**Fig. 2:**
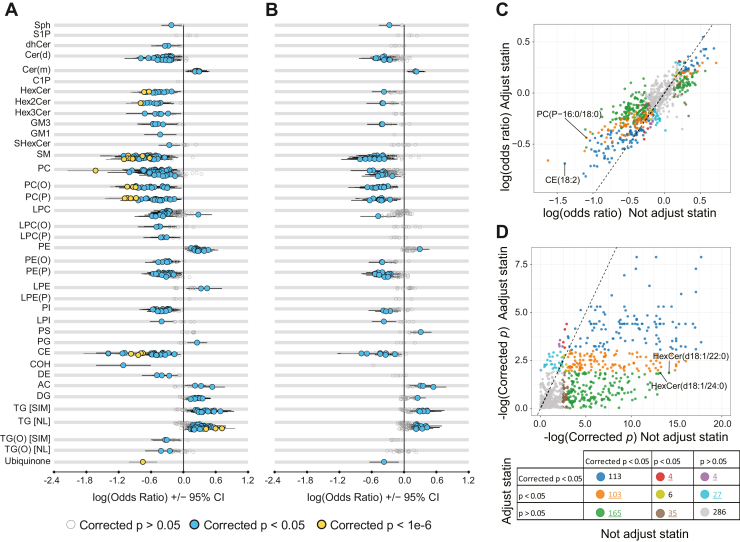
Effect of statin usage adjustment on the association of prevalent CVD with AusDiab lipid species. Logistic regressions were conducted to assess the association of prevalent CVD with lipid concentration while adjusting for age, sex, BMI, hypertension medication, diabetes history, current smoking, blood pressure (A) and the same covariates with statin usage (B). Grey empty circles show species with corrected *P* > 0.05, blue circles show species with corrected *P* < 0.05, yellow circles show species with corrected *p* < 1e-6. Scatter plot of log odds ratio (C) and minus log-transformed corrected *P* values (D) of each lipid with and without adjusting statin usage, dot color indicates the group of *P* values of each lipid as shown in the bottom table. The number of lipids in each *P* value group were given with lipid showing different p group were highlighted with colors and underline. The dashed line represents the line of identity. CI, confidence interval. AC, Acylcarnitine; CE, cholesteryl ester; Cer(d), ceramide; Cer(m), Deoxyceramide; COH, free cholesterol; DE, dehydrocholesterol; DG, diacylglycerol; dhCer, dihydroceramide; GM1, GM1 ganglioside; GM3, GM3 ganglioside; HexCer, monohexosylceramide; Hex2Cer, dihexosylceramide; Hex3Cer, trihexosylceramide; LPC, lysophosphatidylcholine; LPC(O), lysoalkylphosphatidylcholine; LPC(P), lysoalkenylphosphatidylcholine; LPE, lysophosphatidylethanolamine; LPE(P), lysoalkenylphosphatidylethanolamine; LPI, lysophosphatidylinositol; NL, neutral loss; PC, phosphatidylcholine; PC(O), alkylphosphatidylcholine; PC (P), alkenylphosphatidylcholine; PE, phosphatidylethanolamine; PE(O), alkylphosphatidylethanolamine; PE(P), alkenylphosphatidylethanolamine; PG, phosphatidylglycerol; PI, phosphatidylinositol; PS, Phosphatidylserine; SM, sphingomyelin; SIM, single ion monitoring; Sph, sphingosine; S1P, sphingosine-1-phosphate; SHexCer, Sulfatide; TG, triacylglycerol; TG(O), alkyl-diacylglycerol.

Our analysis revealed that inclusion of statin usage as a covariate resulted in significant alterations in the association of lipid species with CVD risk (Fig. 2A, B and supplemental Table S5). For example, after adjustment for statin usage, the log odds ratio of CE(18:2) reduced in absolute strength from −1.39 to −0.69 (Fig. 2C and supplemental Table S5). This coincided with an increase in *P*-values, with many lipids exhibiting higher *P*-values in the statin-adjusted model. For instance, the corrected *P*-value of HexCer(d18:1/22:0) increased from 6.98 × 10^−07^ to 1.59 × 10^−01^ and HexCer(d18:1/24:0) increased from 1.84 × 10^−06^ to 1.59 × 10^−01^ (Fig. 2D and supplemental Table S5).

We categorized the lipid associations into three groups based on their *P*-values (corrected *P* < 0.05, *P* < 0.05, *P* > 0.05). Adjusting for statin usage had a substantial effect on these categorizations: 405 lipid species remained consistently classified, while 338 lipid species changed groups. Most notably, 268 lipids (yellow and green dots in Fig. 2D) were significant in the unadjusted model (corrected *P* < 0.05) but were no longer significant after statin adjustment (corrected *P* > 0.05). Interestingly, eight lipids increased in significance following adjustment for statin usage (red and purple dots in Fig. 2D and supplemental Table S5).

In summary, our findings emphasize the importance of considering statin usage as a covariate to avoid confounding effects when exploring the association between prevalent CVD and lipidome.

### Development of a robust statin prediction model with improved transferability

**Fig. 3:**
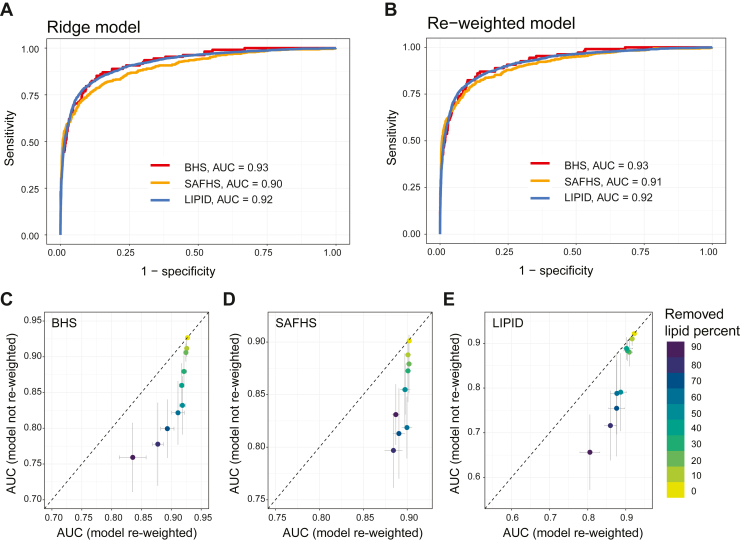
Development of a re-weighted statin usage prediction model with high accuracy, robusticity and transferability. Ridge models and re-weighted models were built using AusDiab lipidome to predict statin usage in BHS, SAFHS and LIPID. AUC plots show similar prediction accuracy in the ridge models (A) and re-weighted models (B). The re-weighted models show higher AUC than ridge models when randomly removing lipids from training and validation cohorts (C–E). When randomly removing lipids, 10 iterations were performed, and the mean and standard deviation were shown. The dashed line represents the line of identity.

Lack of, or incomplete, statin usage information is commonly seen in clinical studies. We tested whether statin usage could be predicted using lipidomic profiles. To this end, we built ridge regression prediction models using the AusDiab lipidome and evaluated the performance of the model in three external cohorts (BHS, SAFHS, and LIPID). The ridge models showed high prediction accuracy with an area under the curve (AUC) greater than 0.9 in all three external cohorts (Fig. 3A) while the model using conventional clinical lipids show much lower AUCs in BHS (AUC = 0.75), SAFHS (AUC = 0.83) and LIPID (AUC = 0.74) (supplemental Fig. S2).

**Fig. 4:**
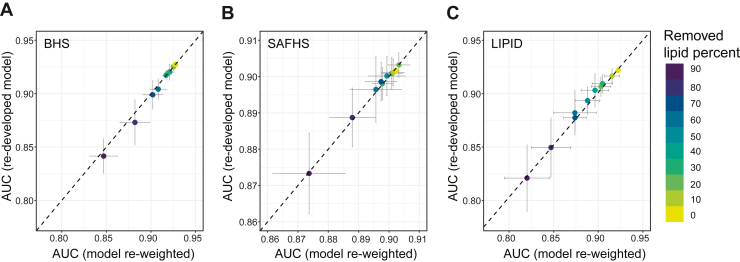
Comparison of the re-weighted models with re-developed ridge models after removing lipids. The re-weighted models were developed using all AusDiab lipids while adjusting for age and sex, and then used to predict statin usage in BHS (A), SAFHS (B) and LIPID (C) cohorts. The ridge model was re-developed each time after randomly removing lipids. When randomly removing lipids, 10 iterations were performed and the mean and standard deviation were shown. The dashed line represents the line of identity.

However, as each validation cohort had a different number of overlapping lipid species with the training cohort, we needed to construct three separate models in AusDiab. To address the limitations, we developed a flexible transfer model (see details in the Materials and Methods section) that allows a single model to be produced in the training cohort, using all available lipid species. Then, when evaluating the model on an external dataset, the beta-coefficients for overlapping variables are re-weighted, allowing seamless transferability of the model to datasets with fewer lipid species. The re-weighted models achieved effectively identical performance compared to the three cohort-specific ridge models in the validation cohorts (Fig. 3B). We then investigated the robustness of the flexible transfer model by artificially reducing the number of overlapping lipids randomly, by up to 90% and comparing the performance of the re-weighted models with the original ridge model where the coefficients for missing lipids would be 0. The re-weighted model shows higher AUC values than the original ridge model in all three external cohorts (Fig. 3C–E). When 90% of lipids were removed, the flexible transfer model still maintained a high AUC, exceeding 0.8 across all three cohorts. Furthermore, we compared the performance of the flexible transfer model to newly developed ridge models using the randomly selected overlapping lipids and saw similar performance (Fig. 4). This shows that the flexible transfer models are accurately reflecting the performance of purpose-built models, without the need for remodeling on the reduced lipid set.

To further compare the performance of the flexible transfer models and the ridge models, we used SAFHS (857 lipids) and LIPID (342 lipids) lipidomic data to construct models and validated the prediction accuracy in AusDiab, BHS, and SAFHS/LIPID. As previously observed, the flexible transfer models showed similar AUC as the cohort-specific ridge models when using all overlapping lipids (supplemental Figs. S3A, B and S4A, B). When there were missing lipids in the validation cohorts, the flexible transfer models outperformed the original ridge model (supplemental Figs. S3C–E and S4C–E). Once more, the flexible transfer model showed similar AUCs compared to newly developed ridge models that accounted for the missing lipids (supplemental Figs. S3F–H and S4F–H), indicating that the re-weighted model not only has comparable prediction accuracy but also better transferability compared to the ridge model.

## Discussion

Statins stand as a widely utilized medication for both the prevention and treatment of CVD. Consistent with earlier findings, our study revealed predominantly negative associations between statin treatment and the concentration of many lipid species, with a select few lipid species demonstrating positive associations (Fig. 1A) (6, 29). Given the influence of statin usage on the lipidome and CVD risk, statin usage becomes a significant confounder for assessing the association between lipid species and CVD risk. To our knowledge, the impact of statin adjustment on the association between the lipidome and disease remains unexplored, despite the substantial implications. In this study, we unveil an amelioration in the association strength of many lipids following the inclusion of statin as a covariate. Notably, we identify 303 putative false-positive and 35 false-negative associations in the model that does not adjust for statin usage (Fig. 2C and supplemental Table S5). These findings robustly advocate for the necessity of statin adjustment when examining the association between diseases/traits and the lipidome.

The mechanisms by which statins exert their effects are largely attributed to their inhibition of cholesterol synthesis and consequential reduction in LDL-C levels—partially mediated by increasing the expression of the low-density lipoprotein receptor (30)—with additional evidence detailing an increase in circulating HDL-C (1, 2, 3, 31). Linear regression analysis between concentrations of the 12-month lipid species and statin usage in the LIPID cohort identified negative associations between statins and most lipid species (Fig. 1A). These findings are consistent with the literature and likely reflect the impact of pravastatin on circulating lipoproteins (32). To explore the influence of statins on lipid metabolism independent of lipoprotein alterations, we repeated the analysis and adjusted for clinical lipids. Results unveiled strong effects on the relative abundance of fatty acids, illustrated by the diverse distribution of lipids positively and negatively associated with pravastatin (Fig. 1B). Lipids containing arachidonic acid (20:4 n-6) and linoleic acid (18:2) were particularly affected by statin usage, with strong positive and negative associations, respectively (supplemental Fig. S5 and supplemental Table S3). These results mirrored work by other groups, detailing extensive fatty acid remodeling in response to simvastatin treatment (33). Statin usage has been shown to influence the sterol regulatory element-binding protein (SREBP) and polyunsaturated fatty acids synthesis pathways, potentially producing the lipid profile identified in this study (29, 34). Furthermore, inhibiting HMGCR activity can lead to elevation of HMG-CoA, which has been proposed to interact with fatty acid synthase, further modulating cellular signaling pathways and lipid metabolism (35). These pleotropic effects of statins, causing substantial modulation of the plasma lipidome, reinforce the confounding effect of statins in lipidomic analyses.

Ridge regression models to predict statin usage, utilizing the AusDiab lipidome (747 lipid species) as the training dataset, demonstrated exceptional performance in BHS, LIPID, and SAFHS, yielding AUC values surpassing 0.9 (Fig. 3A, B). However, when we utilized the less granular lipidomic measures in the LIPID study (342 lipid species), the statin prediction models exhibited diminished performance compared to the AusDiab-based model (supplemental Fig. S4A, B; AUC values of 0.84, 0.72, and 0.74 in AusDiab, BHS, and SAFHS, respectively). Consequently, the model may fail to capture the impact of statin usage in the LIPID study, leading to lower prediction accuracy. This point is supported by the robustness testing of the flexible transfer models, where using fewer lipids leads to decreasing AUC (Fig. 4). For instance, after removing 90% percent of the lipid species from AusDiab, the AUC of the model dropped from 0.93 to 0.84 when validating in BHS (Fig. 4A). Another potential explanation could be differences in the coverage of lipid classes. The LIPID study only profiled lipid species from 24 lipid classes, while AusDiab measured lipid species from 36 lipid classes and SAFHS measured 50 lipid classes. Hence, ensuring comprehensive coverage of lipid species and classes is important for the precision of statin prediction. It is worth noting that this study used lipidomic data from plasma (AusDiab, LIPID, SAFHS) and serum (BHS) samples. Previous study suggested that while the absolute concentration of some metabolites were higher in serum than plasma, the metabolites were overall highly correlated between plasma and serum sample with a mean correlation coefficient  of  0.81 (36). Further to this, similar association was observed between plasma and serum lipidome with Alzheimer’s disease (37). In this study, the statin prediction models were built with the plasma lipidome and then externally validated in cohorts with both plasma and serum (Fig. 3A, B, supplemental Figs. S3A, B and S4A, B). When externally validating our model, the model AUC were similar in BHS which use serum samples and other cohorts using plasma samples, indicating the effect of statin on the plasma and serum lipidome is similar.

Missing data have long been a problem for risk prediction models (38). In a clinical setting, physicians have the unique advantage of being able to directly address missing data for risk prediction, for example, by ordering additional tests, collecting participant/family history, or directly collecting anthropometric measurements. However, it is important to recognize that addressing missing data is not always straightforward. In research environments or retrospective studies, it is often impossible to re-acquire the missing data. Therefore, many studies have focused on missing value imputation as a method to overcome the challenge of missing data (39, 40). While this method works when a large dataset is available and missing data is sparse/missing-at-random, it isn’t suitable for risk prediction on a single individual or when variables are missing for every person.

Lipid species are acted on by a network of intersecting molecular pathways. These pathways affect multiple lipid species/classes, which introduce strong correlations into lipidomic datasets. Therefore, there is a level of redundancy in such lipidomic datasets. When creating prediction models, highly correlated lipid species will ‘compete’ for incorporation into the model. Inclusion of a ridge penalty will allow for some sharing of weights between highly correlated variables. In fact, it is often possible to replace one lipid species with another highly correlated species, without loss in generalizability of the model. When a lipid species isn’t measured, it is often possible to predict its concentration using a linear combination of other species (26). This intrinsic flexibility in lipidomic data allows substantial loss and reweighting of predictors without substantial loss in predictive power.

Lipidomics is a rapidly growing field, both in terms of publications and laboratories but also in terms of analytical techniques and platforms. Even within our own high-throughput platform, we have expanded the coverage of the lipidome from 342 lipid species, as used in the LIPID cohort, through to 857 lipid species measured in the SAFHS cohort. When considering other prevalent lipidomic/metabolomic platforms, the number of measured lipid species extends into the thousands. Due to the various analytical strategies employed, each platform measures a subset of all known lipid species, which will often change over time. Furthermore, it is rare to have substantial overlap in the measured variables between platforms. This is because extremely high-throughput methodologies (such as Biocrates p180) will often sacrifice specificity/resolution of molecular lipid species to decrease run times. While the uniqueness of these platforms can provide new research opportunities, it is a field-wide challenge to develop prediction models that are widely transferable and useful.

Few studies have explored procedures to handle missing data at the time of prediction. A common approach is to perform mean imputation, or to ignore the variable altogether. Currently, this is the predominant strategy where quick and practical solutions are required. For instance, it is common when using large polygenic risk scores to encounter thousands of missing values per individual. Alternative methods have been proposed, where multiple prediction models are created a priori (41), hopefully allowing new data to perfectly overlap with one/multiple of these models. Using a similar principle, our approach requires the prediction model to be specified not as beta-coefficients, but as two matrices. This process allows new beta-coefficients to be estimated using any subset of variables from the original model, without the need for the original dataset. This flexibility holds tremendous promise for improving the transferability of ‘omics risk prediction models.

There are several limitations of this study of note. Firstly, the plasma/serum samples used in this study were stored for a relatively long time (AusDiab, 18 years; BHS, 22 years; LIPID, 21 years; SAFHS, 29 years), which might result in the changes of the metabolites. In fact, not only the storage time have an effect on the lipidome, storage temperature and storage conditions also impact the lipidome (42). Most lipid species show relative good stability under −80 °C with gradual degradation over prolonged periods (43). The degradation is more obvious when the storage temperature increases and/or the samples were frequently accessed (44, 45). In this study, although each cohort has been stored for different lengths, the statin prediction models are still validated well. This means that although there might be differences due to storage, they could be considered minor compared to the effects of statins. Information on statin usage among the cohorts was inconsistent (see details in Materials and Methods section). Detailed medication information was obtained for SAFHS and BHS, while AusDiab (at baseline) relied on a more general questionnaire related to “cholesterol/triglyceride lowering”. However, at the 5-year follow-up survey, it was found that statins accounted for 97.3% (1,221 out of 1,255 instances) of all cholesterol/triglyceride lowering medication, supporting previous reports showing the high usage of statins compared to other lipid lowering medication in Australia (46). Furthermore, the type and dose of statin was not modelled in this study. The LIPID trial participants were randomized to receive a fixed dose of pravastatin (40 mg), while the population cohorts would represent a range of types and dosages. Due to only LIPID cohort contains baseline and post statin treatment lipidomic data, the effect of pravastatin on lipidome were directly assessed in this cohort (Fig. 1). However, a previous study from our group using pitavastatin in a small number of individuals reported similar effect of pitavastatin on lipidome including decrease in linoleic acid species and increase in arachidonic acid species after statin treatment, suggesting that a majority of effects can be attributed to a generalized “statin” effect (supplemental Table S3) (47). More modern types of statins show greater efficacy, allowing lower dosages. As such, in clinical settings, the dose of statins are titrated to achieve a desired LDL-C reduction. Future studies may consider if the type and dose of statins are important quantities to predict and whether they have differing effects on the lipidome.

## Conclusion

Adjusting for statin usage is critical when assessing the association between the lipidome and CVD. Confounding, misattribution of effects, and erroneous interpretation all almost certainly occur if statin adjustment is not considered. Our findings show that statin usage has large effects on the lipidome, even beyond alterations in lipoprotein abundance. We demonstrate that not adjusting for statin usage leads to false positive associations between lipid species and CVD. These findings highlight the importance of adjusting for statin usage. However, missing or unreliable information on statin usage is common in clinical studies, especially in large population cohorts. Here, we demonstrated that statin usage can be accurately predicted using lipidomics data. To enhance the transferability of ‘omic prediction models, we developed a flexible transfer model which allows reweighting of coefficients without individual level data. The flexible transfer shows similar performance with ad hoc models built using the same training dataset. The flexible transfer model can greatly enhance the transferability of prediction models without loss in prediction accuracy.

## Data availability

Because of the participant consent obtained as part of the recruitment process for the used four studies, it is not possible to make data publicly available (including the individual deidentified data). Individual-level AusDiab data are available for analyses through application to the Principal Investigator Professor Jonathan Shaw and the AusDiab Committee (Email: Jonathan.Shaw@baker.edu.au). Individual-level LIPID data are available for analyses through application to the study lead Professor John Simes and the LIPID Investigators (Email: John.Simes@sydney.edu.au). The timeframe for response to such requests is within two months. Individual-level data for the BHS are available under restricted access for bona fide research; access can be obtained through applications to the Busselton Population Medical Research Institute (http://bpmri.org.au/research/database-access.html). The SAFHS data are available from J.E Curran, (Email: joanne.curran@utrgv.edu) via a material transfer agreement for work consistent with the informed consent.

## Code availability

Source code for the Flexible Transfer Models (FTM) R package is available on the GitHub repository (https://github.com/BakerMetabolomics/FlexibleTransferModels).

## Supplemental data

This article contains supplemental data.

## Conflicts of interests

The authors declare that they have no conflicts of interest with the contents of this article.

## Supplementary data

Supp Figures
