# Supplementary material for: Statin effects on the lipidome: Predicting statin usage and implications for cardiovascular risk prediction
Source: J Lipid Res. 2025 Apr 10;66(5):100800. doi: 10.1016/j.jlr.2025.100800 (PMC12139500; doi:10.1016/j.jlr.2025.100800)
Supplement: Supp Figures [file mmc1.pdf]

SUPPLEMENTAL INFORMATION:

Title: Statin Effects on the Lipidome: Predicting Statin Usage and Implications for Cardiovascular Risk Prediction

Authors: Changyu Yi[1,2,3], Kevin Huynh[1,2,3], Yvette Schooneveldt[1,4], Gavriel Olshansky[1,2], Amy Liang[1], Tingting Wang[1,2,3], Habtamu B Beyene[1,2,3,4], Aleksandar Dakic[1], Jingqin Wu[1], Michelle Cinel[1], Natalie A Mellett[1], Gerald F Watts[5,6], Joseph Hung[5], Jennie Hui[7,8], John Beilby[7], Joanne E Curran[9], John Blangero[9], Eric K Moses[10], John Simes[11], Andrew M Tonkin[12], Leonard Kritharides[13,14], David Sullivan[11,15], The LIPID Study Investigators[16], Jonathan E Shaw[1,4], Dianna J Magliano[1,4], Agus Salim[1,2,17], Corey Giles[1,2,3,*], Peter J Meikle[1,2,3,4,*].

[1]Baker Heart and Diabetes Institute, Melbourne, Australia.
[2]Baker Department of Cardiometabolic Health, Melbourne University, Melbourne, Australia.
[3]Baker Department of Cardiovascular Research Translation and Implementation, La Trobe University, Melbourne, Australia.
[4]Faculty of Medicine, Nursing and Health Sciences, Monash University, Melbourne, Australia.
[5]School of Medicine, University of Western Australia, Perth, Australia.
[6]Lipid Disorders Clinic, Department of Cardiology, Royal Perth Hospital, Perth, Australia.
[7]School of Biomedical Sciences, University of Western Australia, Perth, Australia.
[8]School of Population and Global Health, University of Western Australia, Perth, Australia.
[9]South Texas Diabetes and Obesity Institute, The University of Texas Rio Grande Valley, Brownsville, Texas, USA.
[10]Menzies Institute for Medical Research, University of Tasmania, Hobart, Australia.
[11]National Health and Medical Research Council of Australia (NHMRC), Clinical Trials Centre, University of Sydney, Sydney, Australia.
[12]School of Public Health and Preventive Medicine, Monash University, Melbourne, Australia.
[13]Atherosclerosis and Vascular Biology Laboratory, ANZAC Medical Research Institute, Sydney, Australia.
[14]Concord Repatriation General Hospital, Sydney Local Health District, Sydney, Australia.
[15]New South Wales Health Pathology, Sydney, Australia.
[16]A list of authors appears at the Acknowledgements section of the paper.
[17]Melbourne School of Population and Global Health and School of Mathematics and Statistics, The University of Melbourne, Melbourne, Australia.
* Co-senior and corresponding authors.

Corresponding authors:

Core Giles: corey.giles@baker.edu.au

Peter Meikle: peter.meikle@baker.edu.au

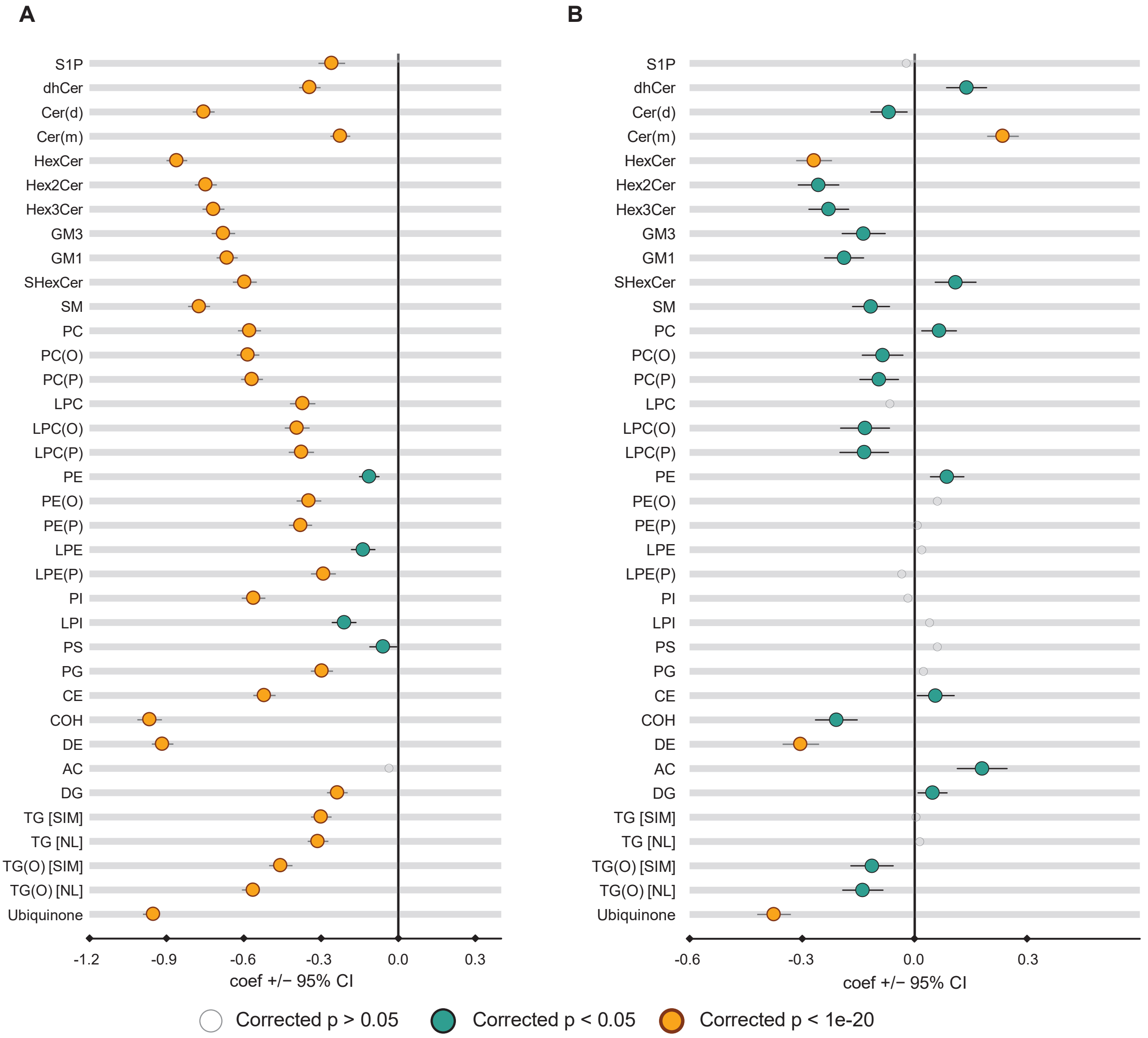

Supplemental Fig. 1, Association of statin usage with LIPID trial lipid classes using linear regression. Linear regression analysis between each 12-month lipid class concentration and statin usage was performed while adjusting for age, sex, BMI, hypertension medication, current smoking, blood pressure, baseline lipid concentration, interaction between baseline lipid concentration, statin usage and clinical lipids (total cholesterol, HDL-C, triglycerides) from baseline (A) as well as clinical lipids from 12-month follow-up (B). Lipid concentrations were log-transformed before performing linear regression. Grey empty circles show lipid classes with corrected $p > 0.05$, dark-green circles show lipid classes with corrected $p < 0.05$, yellow circles show lipid classes with corrected $p <$ 1e-20. Whiskers represent 95% confidence intervals. The underlying data can be found in Supplemental Table 3. AC, Acylcarnitine; CE, cholesteryl ester; Cer(d), ceramide; Cer(m), Deoxyceramide; COH, free cholesterol; DE, dehydrocholesterol; DG, diacylglycerol; dhCer, dihydroceramide; GM1, GM1 ganglioside; GM3, GM3 ganglioside; HexCer, monohexosylceramide; Hex2Cer, dihexosylceramide; Hex3Cer, trihexosylceramide; LPC, lysophosphatidylcholine; LPC(O), lysoalkylphosphatidylcholine; LPC(P), lysoalkenylphosphatidylcholine; LPE, lysophosphatidylethanolamine; LPE(P), lysoalkenylphosphatidylethanolamine; LPI, lysophosphatidylinositol; NL, neutral loss; PC, phosphatidylcholine; PC(O), alkylphosphatidylcholine; PC (P), alkenylphosphatidylcholine; PE, phosphatidylethanolamine; PE(O), alkylphosphatidylethanolamine; PE(P), alkenylphosphatidylethanolamine; PG, phosphatidylglycerol; PI, phosphatidylinositol; PS, Phosphatidylserine; SM, sphingomyelin; SIM, single ion monitoring; S1P, sphingosine-1-phosphate; SHexCer, Sulfatide; TG, triacylglycerol; TG(O), alkyl-diacylglycerol.

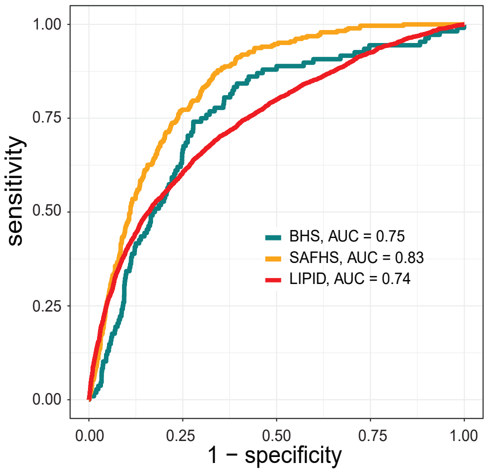

Supplemental Figure 2, Prediction of statin usage with clinical lipids. A logistic regression model was built using AusDiab clinical lipids (total cholesterol, HDL-C and triglycerides) while adjusting for age and sex. The model was applied to BHS, SAFHS and LIPID datasets to predict statin usage and the AUC were calculated.

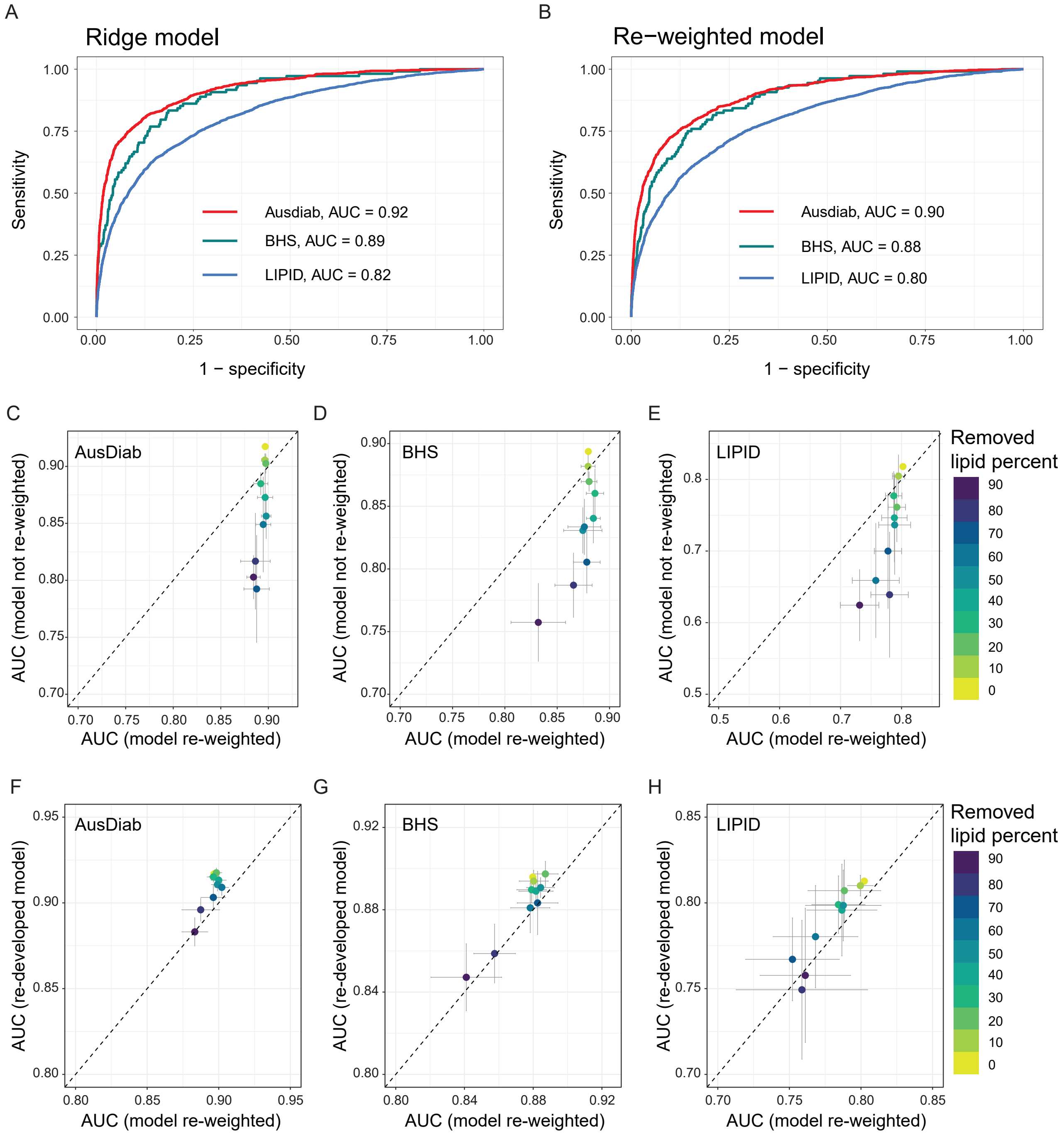

Supplemental Figure 3, Development of a re-weighted statin usage prediction model with SAFHS lipidome. Ridge model and re-weighted model were built using SAFHS lipidome to predict statin usage in AusDiab, BHS and LIPID. Area under the curve (AUC) plots show similar prediction accuracy in the ridge model (A) and re-weighted model (B). The re-weighted model shows higher AUC than ridge model AUC after randomly removing lipids (C-E). The re-weighted model shows similar performance with the re-developed ridge model which using the same lipids that were used in the re-weighted model after lipids were removed (F-H). When randomly removing lipids, 10 iterations were performed and the mean and standard deviation were shown. The dashed line represents the line of identity.

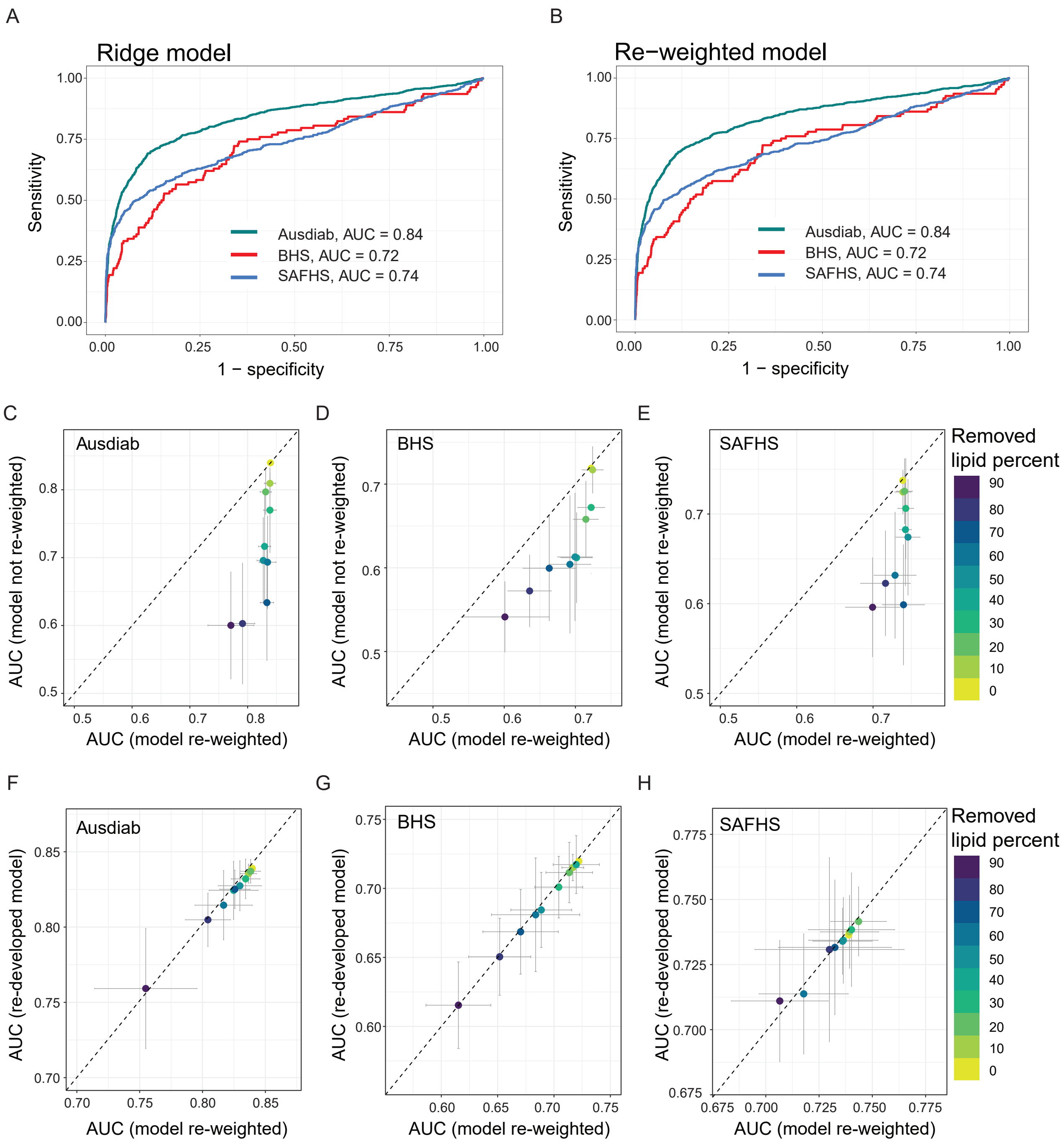

Supplemental Figure 4, Development of a re-weighted statin usage prediction model with LIPID lipidome. Ridge model and re-weighted model were built using LIPID lipidome to predict statin usage in AusDiab, BHS and SAFHS. Area under the curve (AUC) plots show similar prediction accuracy in the ridge model (A) and re-weighted model (B). The re-weighted model shows higher AUC than ridge model AUC after randomly removing lipids (C-E). The re-weighted model shows similar performance with the re-developed ridge model which using the same lipids that were used in the re-weighted model after lipids were removed (F-H). When randomly removing lipids, 10 iterations were performed and the mean and standard deviation were shown. The dashed line represents the line of identity.

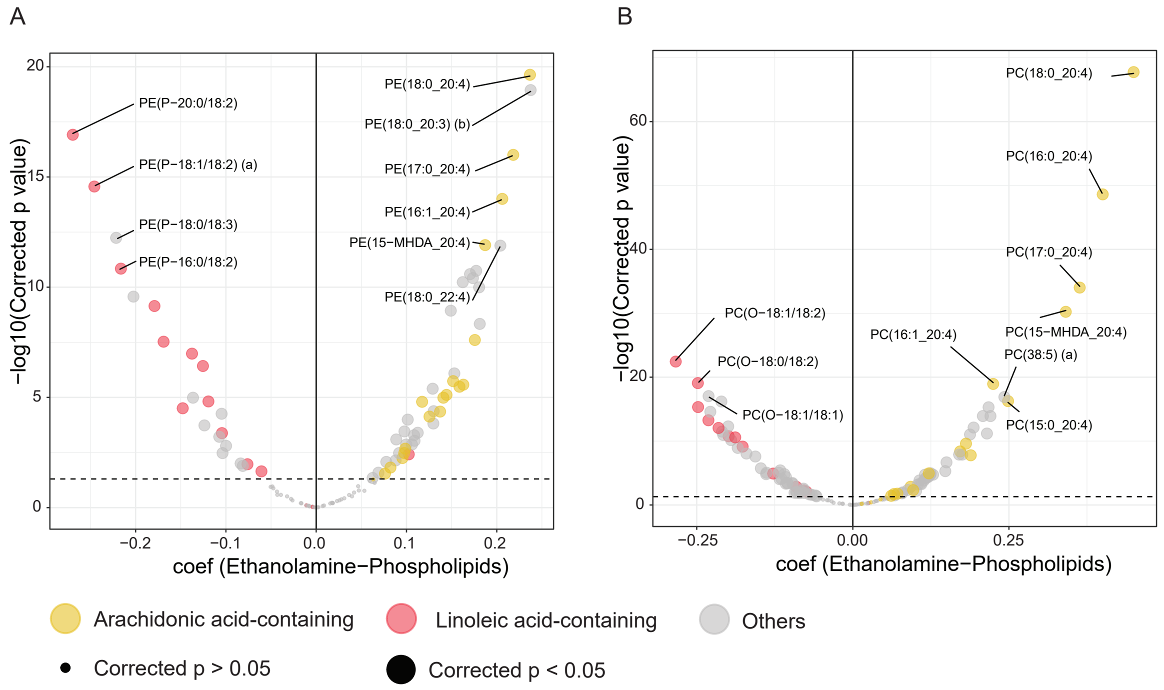

Supplemental Figure 5, Effect of statin treatment on ethanolamine containing phospholipids and choline containing phospholipids. Linear regression analysis was performed between 12-month lipid species and statin usage, adjusting for age, sex, BMI, hypertension medication, current smoking, blood pressure, baseline lipid concentration, baseline and 12-month clinical lipids (total cholesterol, HDL-C, triglycerides) and interaction between baseline lipid concentration, statin usage. (A), ethanolamine containing phospholipid species. (B) choline containing phospholipid species. *p*-values were corrected for multiple comparisons using Benjamini and Hochberg correction. Yellow circles show arachidonic acid-containing species, red circles show linoleic acid-containing, grey circles show other species, and top 10 species based on corrected *p* values were labeled. The dashed horizontal line represents -log10(0.05). Ethanolamine Species; phosphatidylethanolamine [PE], alkyl-phosphatidylethanolamine [PE(O)] and alkenyl-phosphatidylethanolamine [PE(P)], Choline Species; phosphatidylcholine [PC], alkyl-phosphatidylcholine [PC(O)] and alkenyl-phosphatidylcholine [PC(P)].
